# RRAM characteristics using a new Cr/GdO_x_/TiN structure

**DOI:** 10.1186/1556-276X-9-680

**Published:** 2014-12-17

**Authors:** Debanjan Jana, Mrinmoy Dutta, Subhranu Samanta, Siddheswar Maikap

**Affiliations:** Thin Film Nano Technology Laboratory, Department of Electronic Engineering, Chang Gung University, 259 Wen-Hwa 1st Rd., Kwei-Shan, Tao-Yuan 333 Taiwan

**Keywords:** RRAM, GdO_x_, Cr, Resistive switching, Memory

## Abstract

Resistive random access memory (RRAM) characteristics using a new Cr/GdO_x_/TiN structure with different device sizes ranging from 0.4 × 0.4 to 8 × 8 μm^2^ have been reported in this study. Polycrystalline GdO_x_ film with a thickness of 17 nm and a small via-hole size of 0.4 μm are observed by a transmission electron microscope (TEM) image. All elements and GdO_x_ film are confirmed by energy dispersive X-ray spectroscopy and X-ray photoelectron spectroscopy analyses. Repeatable resistive switching characteristics at a current compliance (CC) of 300 μA and low operating voltage of ±4 V are observed. The switching mechanism is based on the oxygen vacancy filament formation/rupture through GdO_x_ grain boundaries under external bias. After measuring 50 RRAM devices randomly, the 8-μm devices exhibit superior resistive switching characteristics than those of the 0.4-μm devices owing to higher recombination rate of oxygen with remaining conducting filament in the GdO_x_ film as well as larger interface area, even with a thinner GdO_x_ film of 9 nm. The GdO_x_ film thickness dependence RRAM characteristics have been discussed also. Memory device shows repeatable 100 switching cycles, good device-to-device uniformity with a switching yield of approximately 80%, long read endurance of >10^5^ cycles, and good data retention of >3 × 10^4^ s at a CC of 300 μA.

## Background

Recently, resistive random access memory (RRAM) is one of the most potential candidates for future nanoscale non-volatile memory application [1–4]. Under external bias, the resistive switching phenomena have been observed in various types of materials including HfO_x_ [5–10], TaO_x_ [11–16], AlO_x_ [17–20], and so on. Besides binary oxides, some rare-earth materials such as yttrium-oxide (Yb_2_O_3_) [21] and gadolinium-oxide (Gd_2_O_3_) [22–27] also attract to the researchers for high-performance RRAM application. However, the Gd_2_O_3_ is one of the promising materials because of its higher energy bandgap 5.4 eV [28], higher dielectric constant 14 to 20 [28, 29], and good chemical and thermal stability [24]. Most importantly, this can form Gd:Gd_2_O_3_ film [22] as well as polycrystalline [22], which will help to have controllable oxygen vacancy filament formation/rupture under external bias. Although, the Gd_2_O_3_ material is useful, however, RRAM properties have been reported infrequently. Aratani et al. [23] have reported the conductive bridging RRAM using Cu or a Ag/CuTe/Gd_2_O_3_/W structure with an operating current of 100 μA. Cao et al. [24] have reported unipolar resistive switching using a Pt/Gd_2_O_3_/Pt structure at high RESET current of approximately 35 mA. Zhou et al. [25] have reported bipolar resistive switching phenomena using a Pt/GdO_x_/TaN_x_ structure at high current compliance (CC) of 1 mA, and high forming voltage is required to switch the device initially. Wang et al. [26] have also reported resistive switching using a Pt-Al/Gd_2_O_3_/W structure at a high CC of 1 mA. Yoon et al. [27] have reported Cu doped MoO_x_/GdO_x_ bilayer resistive switching characteristics with a CC of 300 μA. In our previous study [22], we have reported self-compliance resistive switching phenomena using IrO_x_/GdO_x_/W cross-point structure at a CC of >300 μA. The resistive switching phenomena using a IrO_x_/GdO_x_/W via-hole structure at a high CC of >1 mA have also been reported [30]. Generally, resistive switching characteristics of other RRAMs using binary oxides show high current operation [6, 13, 18], and it is reported rare at low current operation [7, 8, 14]. Further, many electrodes such as TiN, Pt, Ir, IrO_2_, W, Cu, and so on have been also used and known to have high-performance RRAMs; however, the chromium (Cr) in a Cr/Gd_2_O_3_/TiN structure has not been reported yet. The work function of Cr is 4.5 eV [31], which is larger than Al of 4.28 eV [31]. Gibbs free energies of Cr_2_O_3_ and Gd_2_O_3_ are reported -694.88 [32, 33] and -1,730 [34] kJ/mole respectively at 300 K. Therefore, the Cr will not be oxidized easily with respect to Gd_2_O_3_ switching material. This is benefited of Cr in the Cr/Gd_2_O_3_/TiN structure.

In this study, repeatable bipolar resistive switching characteristics of the Cr/GdO_x_/TiN RRAM devices at a CC of 300 μA and low operating voltage of ±4 V have been investigated for the first time. Polycrystalline GdO_x_ film and a via-hole size of 0.4 μm are observed by both the transmission electron microscope (TEM) and energy-dispersive X-ray spectroscopy (EDS) analysis. The resistive switching phenomena with variation of device sizes ranging from 0.4 × 0.4 to 8 × 8 μm^2^ have been discussed. More than 50 randomly picked devices are measured. Large size devices (8 μm) show superior resistive switching characteristics as compared to those of the small size devices (0.4 μm) at a CC of 300 μA. Memory device shows good 100 switching cycles, device-to-device uniformity, program/erase (P/E) endurance of >100 cycles, and long read endurance of >10^5^ cycles. Memory device also shows excellent data retention of more than 3 × 10^4^ s with a large resistance ratio of >70.

## Methods

**Figure 1:**
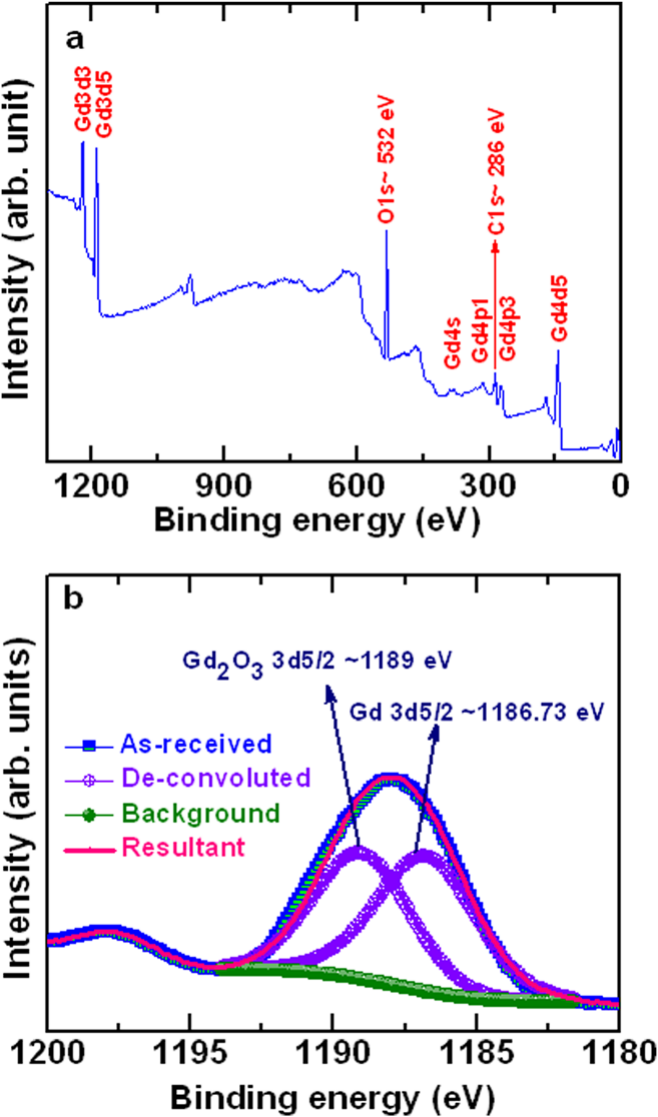
**XPS characteristics. (a)** Broad and **(b)** narrow scan spectra. The Gd-rich GdO_x_ film is confirmed. The thickness of the GdO_x_ film was 17 nm.

The Cr/GdO_x_/TiN RRAM devices were fabricated as follows. First, the SiO_2_ layer with a thickness of 200 nm was deposited on an 8-in Si substrate. Then, TiN as a bottom electrode (BE) was deposited on an SiO_2_/Si substrate. The thickness of TiN BE was approximately 200 nm. In next step, an SiO_2_ layer with a thickness of 150 nm was deposited on TiN BE. Then, the via-holes with different sizes ranging from 0.4 × 0.4 to 8 × 8 μm^2^ and BE contacts were designed and etched. Photo-resist was coated and patterned for switching material and the top electrode (TE) contacts. Therefore, another lithography step was used to pattern the devices for lift-off. After that, a small piece of approximately 1 × 1 in^2^ was cut from the 8-in patterned wafer and deposited consecutive switching material and the top electrode. The Gd_2_O_3_ as a resistive switching material was deposited by an electron-beam evaporation method. Pure Gd_2_O_3_ shots were used during evaporation. The deposition rate of Gd_2_O_3_ was 0.2 Å/s, and the power was 400 W. After deposition, the Gd_2_O_3_ material was a Gd-rich Gd_2_O_3_ film which was confirmed by X-ray photo-electron spectroscopy (XPS) analysis [22]. Broad scan of XP spectra is shown in Figure 1a. The Gd (3d, 4 s, 4p, and 4d), O1s, and C1s peaks are also observed. XPS spectra of Gd 3d_5/2_ and Gd_2_O_3_ 3d_5/2_ peaks were located at 1186.73 eV and 1,189 eV, respectively, which confirmed a Gd-rich Gd_2_O_3_ film, i.e., GdO_x_ (Figure 1b). The area ratio in between Gd and Gd_2_O_3_ is 1:0.89. This suggests that the as-deposited Gd_2_O_3_ film is a Gd-rich GdO_x_ film. Then, the Cr TE was deposited by rf sputtering process. Argon (Ar) gas flow rate was 10 sccm during deposition. The deposition power and chamber pressure were 100 W and 6 mTorr, respectively. Finally, a lift-off process was performed to get the final RRAM device. The thickness of the GdO_x_ film was 17 nm. For comparison, the thickness of the GdO_x_ film was also 9 nm. Microstructure of a Gd_2_O_3_ film in the RRAM devices was carried out by using TEM-JEOL 2100 F system (JEOL Ltd., Akishima-shi, Japan) with energy of 200 keV and resolution of 0.2 nm. Memory characteristics were performed by using HP 4156C precision parameter analyzer system (Agilent Technologies, Inc., Santa Clara, CA, USA). During electrical measurement of the memory devices, the BE was grounded and the sweeping bias was applied on the TE. All measurements were characterized inside the black box on an 8-in chuck.

## Results and discussion

**Figure 2:**
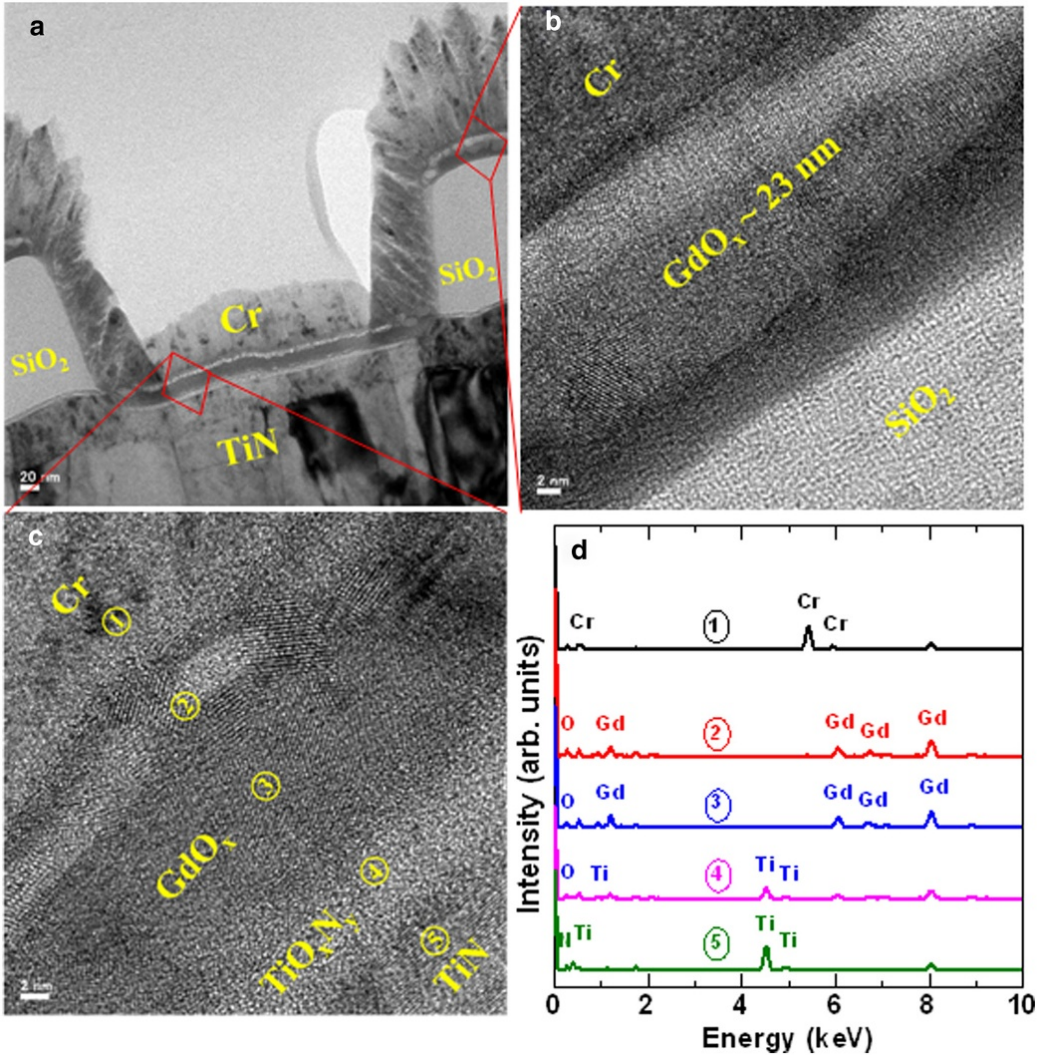
**TEM and EDS analysis. (a)** TEM image of our Cr/GdO_x_/TiN RRAM device. Device size is 0.4 × 0.4 μm^2^. HRTEM image of Cr/GdO_x_/TiN memory device at **(b)** the outside and **(c)** the inside of the via-hole regions. The thicknesses of the GdO_x_ layer at the outside and inside of via holes are 23 and 17 nm, respectively. **(d)** Energy dispersive X-ray spectra (EDS) show Cr, Gd, Ti, N, and O elements. The positions of all spectra taken from TEM image are shown in **(c)**.

**Figure 3:**
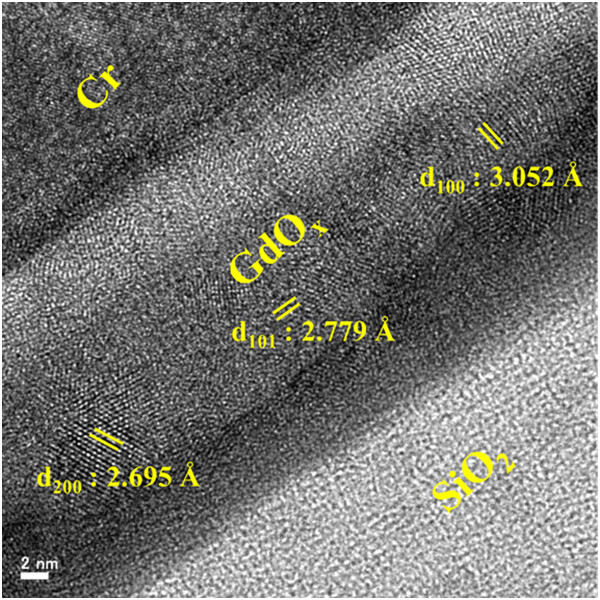
**HRTEM image.** Polycrystalline grains in the GdO_x_ films are shown. It is a large view of Figure 2b.

Figure 2a shows the TEM image of the Cr/GdO_x_/TiN RRAM device. Device size is approximately 0.4 × 0.4 μm^2^. High-resolution TEM (HRTEM) images at the outside and inside of the via-hole regions are shown in Figure 2b,c, respectively. It is observed that the thickness of the GdO_x_ layer is higher at the outside as compared to the inside regions (23 vs. 17 nm). This occurs owing to the physical vapor deposition method. The thickness of Cr is approximately 70 nm inside the via-hole region. Another layer of TiO_x_ (i.e., TiO_x_N_y_) with a thickness of approximately 3 nm inside the via-hole region is observed, as shown in Figure 2c. This is due to the fact that Ti is more reactive with O_2_ (-888 kJ/mole at 300 K [35]), which results in formation of TiO_x_N_y_ layer at the GdO_x_/TiN interface. Figure 2d represents EDS spectra of the Cr/GdO_x_/TiN RRAM device. The EDS spectra correlates with positions 1, 2, 3, 4, and 5, as shown in Figure 2c, which confirms the presence of Cr, Gd, Ti, O, and N elements in the respective layers. The kinetic energy values of Cr, Gd, Ti, O, and N at maximum peak positions are found to be 5.42, 8.04, 4.52, 0.26, and 0.28 eV, respectively, which are similar to the reported energy values [36–38]. The weight and atomic percentages of each element in each position of Figure 2c have been described in Table 1. From positions 2 and 3, it is observed that the GdO_x_ layer is separated into two sub-layers. The values of weight percentage of O in positions 2 and 3 are 13.3% and 11.2% whereas atomic percentages are 53.1% and 51.6%, respectively. Therefore, the oxygen content is slightly lower at position 3 than that at position 2. This represents that the ‘oxygen-rich’ GdO_x_ layer with a thickness approximately 3 nm is formed at the TE/GdO_x_ interface (i.e., white region at the TE side). It is known that Gibbs free energy of Cr_2_O_3_ and Gd_2_O_3_ are -694.88 [32, 33] and -1,730 [34] kJ/mole, respectively. During Cr deposition by a sputtering process, it might be possible that few oxygen ions (O^2-^) from the Gd_2_O_3_ film move towards TE to form Cr_2_O_3_. According to lower Gibbs free energy of Cr_2_O_3_ comparing with Gd_2_O_3_, Cr might not be oxidized and O^2-^ ions accumulate at the TE/GdO_x_ interface having formation of oxygen-rich and oxygen-deficient GdO_x_ layers, respectively, as shown in Figure 2c. It is observed that the thickness of GdO_x_ layer at the outside via region is approximately 23 nm (Figure 2b), which is higher than the thickness of GdO_x_ layer at the inside via-hole region (Figure 2c). Therefore, the crystallinity of the GdO_x_ film at the outside region will be more as well as different crystal orientation could be observed, which results to another layer being observed at the Gd_2_O_3_/SiO_2_ interface. The calculated d spacing is 2.695(d_200_), 2.779(d_101_), or 3.052 Å (d_100_) which confirms the GdO_x_ film being polycrystalline, as shown clearly in Figure 3. Crystal grains in the GdO_x_ films are also reported previously [22]. Li et al. [28] also reported the polycrystalline Gd_2_O_3_ film deposited by sputtering. This suggests that the Gd_2_O_3_ film is polycrystalline in nature, which will have weak bonds on the grain boundary sites and lead to the repeatable resistive switching memory characteristics.

**Table 1:** **Weight and atomic percentages**

EDX spectra taken in Figure 2c	Element	Weight (%)	Atomic (%)
1	Cr	96.5	93.7
2	Gd	80.5	32.7
	O	13.3	53.1
3	Gd	84.9	39.8
	O	11.2	51.6
4	Ti	38.2	40.2
	O	12.0	37.7
5	Ti	70.5	43.6
	N	23.9	50.5

All elements in the Cr/GdO_x_/TiN RRAM device at different positions, as marked in Figure 2c, are shown.

**Figure 4:**
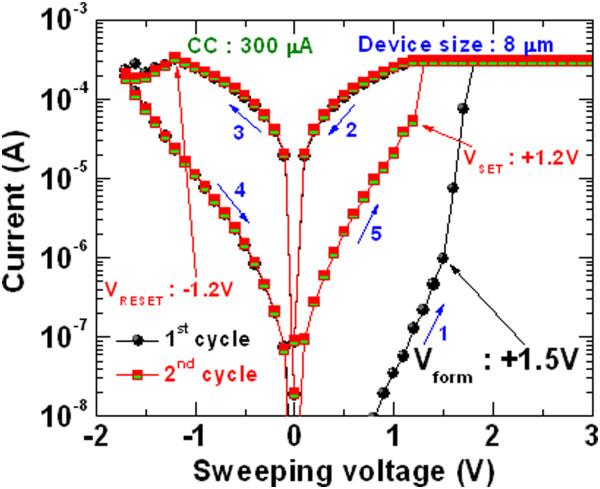
**Current-voltage characteristics of the RRAM devices.** Bipolar *I-V* characteristics of our memory device with a size of 8 × 8 μm^2^ and a GdO_x_ film thickness of 17 nm. The memory device operates under a small operating voltage of ±2 V, and a CC of 300 μA is used.

**Figure 5:**
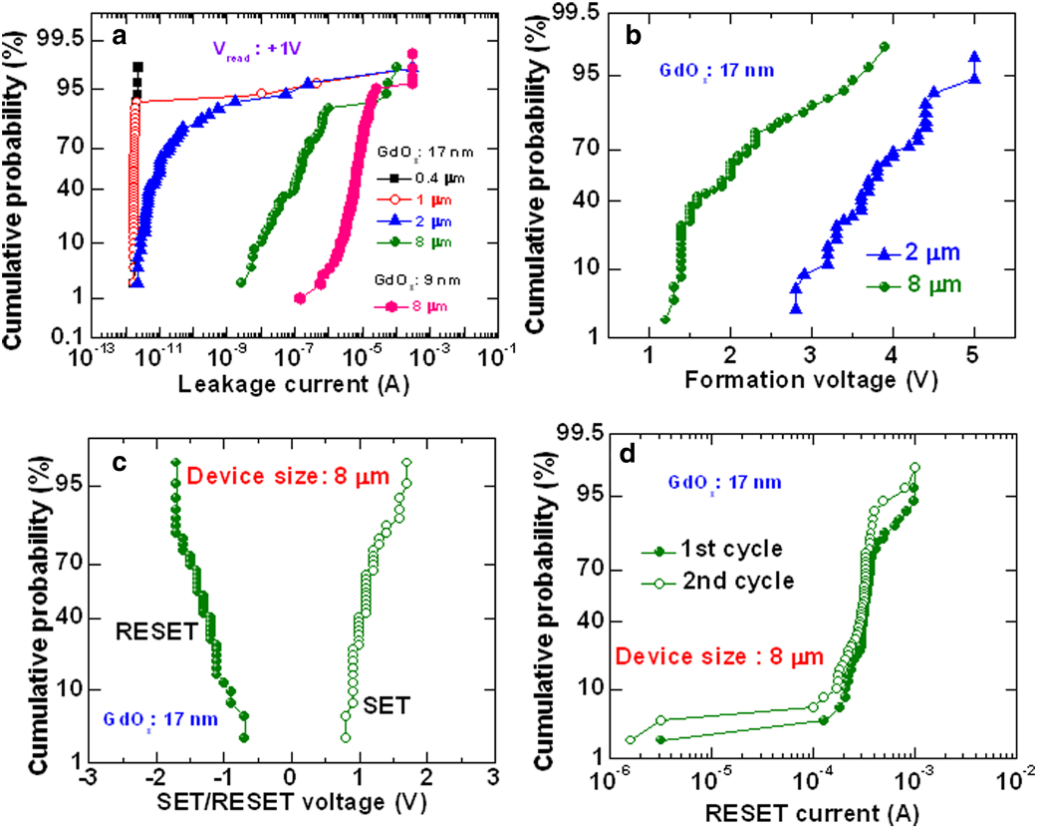
**Cumulative probability of leakage current, formation voltage, SET/RESET voltage, and RESET currents. (a)** Leakage current distributions with different device sizes ranging from 0.4 × 0.4 to 8 × 8 μm^2^. The thicknesses of GdO_x_ film are 17 and 9 nm. **(b)** Forming voltage, **(c)** SET/RESET voltage, and **(d)** RESET currents with different device sizes and a thickness of GdO_x_ film of 17 nm. Fifty devices were measured randomly for each size. It is found that the 8-μm RRAM device shows best uniformity as compared to other sizes.

**Figure 6:**
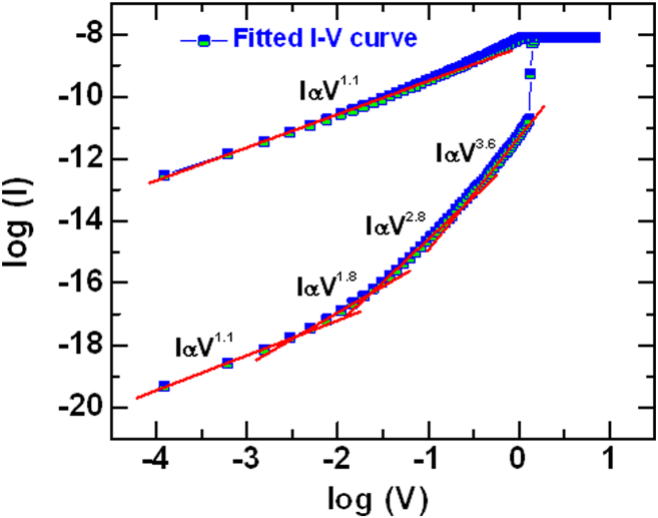
***I-V*** **fitting and carrier transport mechanism.** *I-V* curve fitted in log-log scale. It is found that the HRS is TC-SCLC and LRS is ohmic conduction.

Figure 4 exhibits typical bipolar current-voltage (*I-V*) characteristics of the Cr/GdO_x_/TiN RRAM device with a size of 8 × 8 μm^2^. The thickness of the GdO_x_ film is 17 nm. The sweeping voltage is shown, as indicated by 1 to 5 inside the figure. This RRAM device is operated with a CC of 300 μA. First switching cycle of the memory device shows low formation voltage (*V*_form_) +1.5 V. Initially, memory devices show low leakage current, which is controlled by the size of the device, and defects and thickness of the GdO_x_ film. Figure 5a represents cumulative probability of the leakage currents of randomly measured more than 50 RRAM devices with sizes ranging from 0.4 × 0.4 to 8 × 8 μm^2^. It is observed that the leakage current increases with increasing device sizes from 2 to 8 μm. A large size device has more defects than that of a smaller device. That is why the 8-sμm devices have the highest leakage current. On the other hand, the leakage currents are the same for the 1- and 0.4-μm devices, which is due to the current measurement limitation by our probe station. The leakage current is increased by decreasing the thickness of the switching layer of 9 nm, as shown in Figure 5a. Basically, both the smaller device size and the thicker GdO_x_ film of 17 nm have smaller leakage current. As similar to the device size dependent leakage current, the *V*_form_ also decreases with increasing the device sizes. Figure 5b represents the distribution of the formation voltages of more than 50 RRAM devices. The average values of *V*_form_ are found to be 3.5 and 1.9 V for the 2- and 8-μm devices, respectively. However, the average SET voltage (*V*_SET_) has little changes from 1.27 to 1.12 V for the 2- to 8-μm devices (Figure 5c). Therefore, the *V*_SET_ is independent of the device sizes from 2 to 8 μm. This indicates that all 50 devices with size of 8 μm can be operated at a low voltage of <4 V, which would be very useful for practical realization. It is also observed that all 8-μm devices show formation (yield of 100%) whereas the 2-μm devices have only 72% yield. Even after formation, the clear SET is observed only 40% of 2-μm devices. Therefore, some devices do not show RESET. However, the clear SET is observed 78% of the 8-μm devices. The 8-μm device shows a typical *V*_SET_ (1.2 V) from the second cycle, as shown in Figure 4. After that, the memory device shows good bipolar resistive switching phenomena under small RESET voltage (*V*_RESET_) of -1.2 V. The average *V*_RESET_ value of 50 devices is found to be -1.5 V (Figure 5c). The value of average *V*_RESET_ is similar or higher than the value of *V*_SET_, which is useful for better read operation of these RRAM devices. Even this RRAM device can read at negative voltage because of the higher *V*_RESET_ values. In Figure 4, the RESET current (*I*_RESET_) is found to be 320 μA. This suggests that both SET and RESET currents (300 vs. 320 μA) are almost the same which signifies good current clamping between two electrodes and GdO_x_ switching material. Considering 50 RRAM devices with a size of 8 μm (Figure 5d), the average value of *I*_RESET_ is higher for the first cycle as compared to the second cycle (320 vs. 390 μA), which is owing to a current overshoot effect during the formation or the first cycle of the pristine device at a CC of 300 μA. However, most of the devices show slightly higher *I*_RESET_ for the first cycle. The current conduction is understood by fitting an *I-V* curve in a log-log scale, as shown in Figure 6. Slope value of current at a low resistance state (LRS) is 1.1 (IαV^1.1^) whereas slope values of current at a high resistance state (HRS) are 1.1 (IαV^1.1^), 1.8 (IαV^1.8^), 2.8 (IαV^2.8^), and 3.6 (IαV^3.6^) at low to high voltage regions, respectively. The slope values of HRS are reported 1, 2, 4, and 6 by Shang et al. [39], 1.1, 1.3, and 8.5 by Rubi et al. [40], and 1.2, 2.2, and 3.9 by us [41]. This represents that the current transport of LRS is dominating by Ohmic whereas HRS follows by trap controlled space charge limited current conduction (TC-SCLC) of our RRAM device. The resistive switching mechanism is based on the formation and rupture of oxygen vacancy conducting filament in the GdO_x_ material depending upon electrical stimulus. When positive bias is applied on the TE, the weak Gd-O bonds on the grain boundaries break and oxygen ions (O^2-^) migrate towards the TE and leaving behind oxygen vacancy as well as conducting path formed through polycrystalline grain boundary. Then, the memory device triggers from HRS to LRS. Considering the Gibbs free energy of Cr_2_O_3_ and Gd_2_O_3_, the Cr TE is not oxidized and a part of GdO_x_ is shown to be oxygen-rich (Figure 2). Basically, the oxygen vacancy filament is formed in between the O-rich GdO_x_ and TiO_x_N_y_ layers. The oxygen vacancy filaments in different switching materials are also reported by other groups [1, 5, 7]. Both O-rich GdO_x_ and TiO_x_N_y_ interfacial layers will behave as a series of the conducting filaments, and a current overshoot effect is not observed (Figure 5d). When a negative voltage is applied on the TE, O^2-^ ions are driven out from TE/GdO_x_ interface and re-oxidize the conductive path and memory device switch back from LRS to HRS. Therefore, the O^2-^ ions migrate through crystal grain boundaries and will control the SET/RESET of both the resistance states.

**Figure 7:**
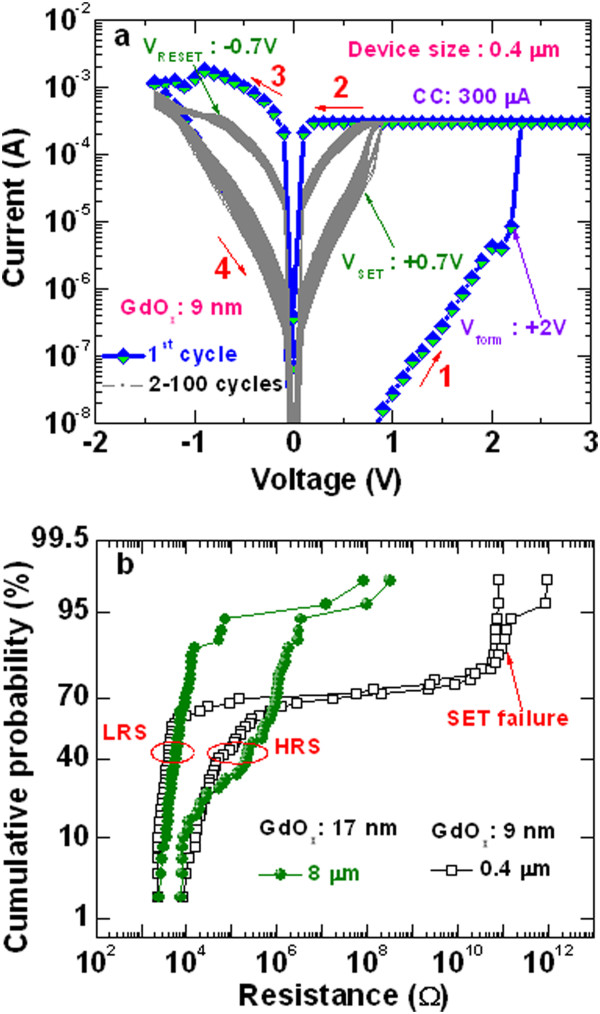
**Repeatable** ***I-V*** **characteristics and cumulative probability of HRS and LRS. (a)** Hundred *I-V* characteristics of the 0.4-μm devices. **(b)** Statistical distributions of HRS and LRS for the 8- and 0.4-μm devices are plotted. Fifty devices were measured randomly. The thicknesses of the GdO_x_ film were 17 and 9 nm for the 8- and 0.4-μm devices, respectively. By considering resistance ratio of >2, successful devices are found to be 78% and 72% for the 8- and 0.4-μm devices, respectively.

It is observed that the 0.4-μm devices with thicker a GdO_x_ film of 17 nm do not show formation as well as resistive switching phenomena owing to thicker switching layer and smaller active area. By reducing thickness of the GdO_x_ film up to 9 nm, the clear formation and SET operation could be observed even at a smallest size of 0.4 μm in our process. Figure 7a illustrates typical bipolar *I-V* characteristics for a device size of 0.4 × 0.4 μm^2^. The device performs consecutive 100 dc cycles with less distribution of LRS and HRS under a CC of 300 μA. The values of *V*_form_, *V*_SET_, and *V*_RESET_ are found to be 2 V, 0.7 V, and -0.7 V, respectively. The values of *I*_RESET_ are found to be 1.8 mA and 375 μA for the first and second cycles, respectively. After measuring 100 RRAM devices, the values of *V*_form_, *V*_SET_, *V*_RESET_, and *I*_RESET_ (first/second cycle) at 50% probability are found to be 1.7 V, 0.9 V, and -0.7 V, and 1.07 mA/391 μA for the 8-μm devices, and those values are found to be 2.5 V, 0.7 V, and -0.8 V, and 1.35 mA/370 μA for the 0.4-μm devices, respectively (not shown here). Therefore, the 8-μm devices have lower formation voltage and smaller RESET current at the first cycle as compared to the 0.4-μm devices, which suggests that larger size devices have a better performance even with the thinner GdO_x_ film of 9 nm. To check the uniformity of the resistance states, we have measured randomly >50 devices and studied statistical distribution of HRS and LRS of device-to-device with device sizes of 0.4 and 8 μm, as shown in Figure 7b. The thickness of the GdO_x_ film is 17 nm for the 8-μm devices and 9 nm for the 0.4-μm devices. Except for few devices which have a small resistance ratio (HRS/LRS) of <2, it is found that the 8-μm device shows better device-to-device uniformity with a high yield >78% as compared to the 0.4-μm devices with a yield >72%. The 8-μm device with a 9-nm-thick GdO_x_ film has also a high yield >88% (not shown here). Further, the 0.4-μm devices show SET failure (Figure 7b), which is reported similar in literature [42]. The values of HRS and LRS for the 8-μm devices at 50% probability are 471.6 and 6.6 kΩ, whereas those values are 126.58 and 4.52 kΩ for the 0.4-μm devices, respectively. The value of LRS is lower for the 0.4-μm devices than those of the 8-μm devices, which is due to higher *I*_RESET_. Therefore, it is observed that the 8-μm device exhibits better uniformity and resistance ratio as compared to the 0.4-μm device. This suggests that recombination rate of oxygen ion (O^2-^) with oxygen vacancy filament is less due to a smaller TE/GdO_x_ interface area for the 0.4-μm devices. So dissolution of oxygen vacancy filament is less for the 0.4-μm devices resulting in higher RESET current, lower resistance ratio, and poor device-to-device uniformity. In the case of the 8-μm devices, recombination rate of oxygen ion (O^2-^) with oxygen vacancy filament is higher due to a larger TE/GdO_x_ interface area, which results in lower RESET current, higher resistance ratio, and better device-to-device uniformity. Chen et al. [43] reported the oxygen recombination rate dependence improved resistive switching characteristics using HfO_x_-based RRAMs. The larger interface area has better switching characteristics because of a higher oxygen recombination rate. This implies that the TE/GdO_x_ interface area in the Cr/GdO_x_/TiN structures plays an important role to have superior switching phenomena. Further study is also needed to unravel the effect of switching performance on different thicknesses of the GdO_x_ layer.

**Figure 8:**
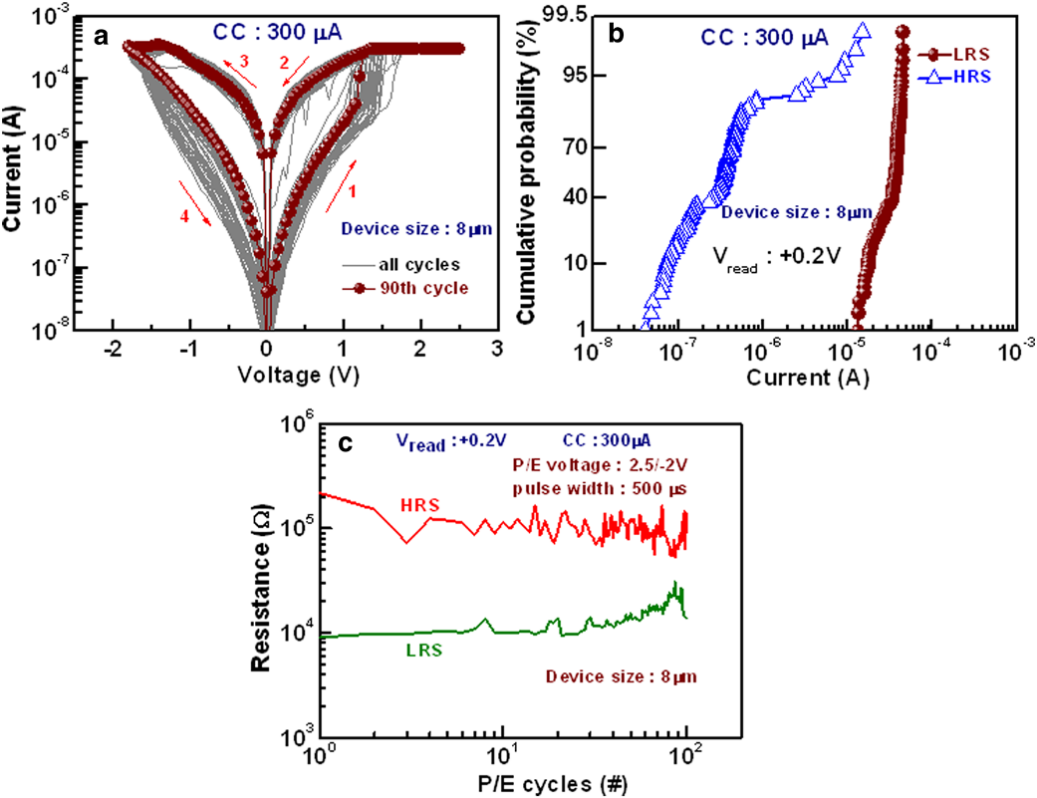
**Switching cycle-to-cycle uniformity. (a)** Repeatable 90 *I-V* switching cycles are shown for a GdO_x_ film thickness of 17 nm. The *V*_SET_ is varied from 1 to 1.5 V and *V*_RESET_ is about -1.5 V. **(b)** Hundred cycle-to-cycle statistical distribution of currents at HRS and LRS. **(c)** The P/E endurance of >100 cycles is obtained.

**Figure 9:**
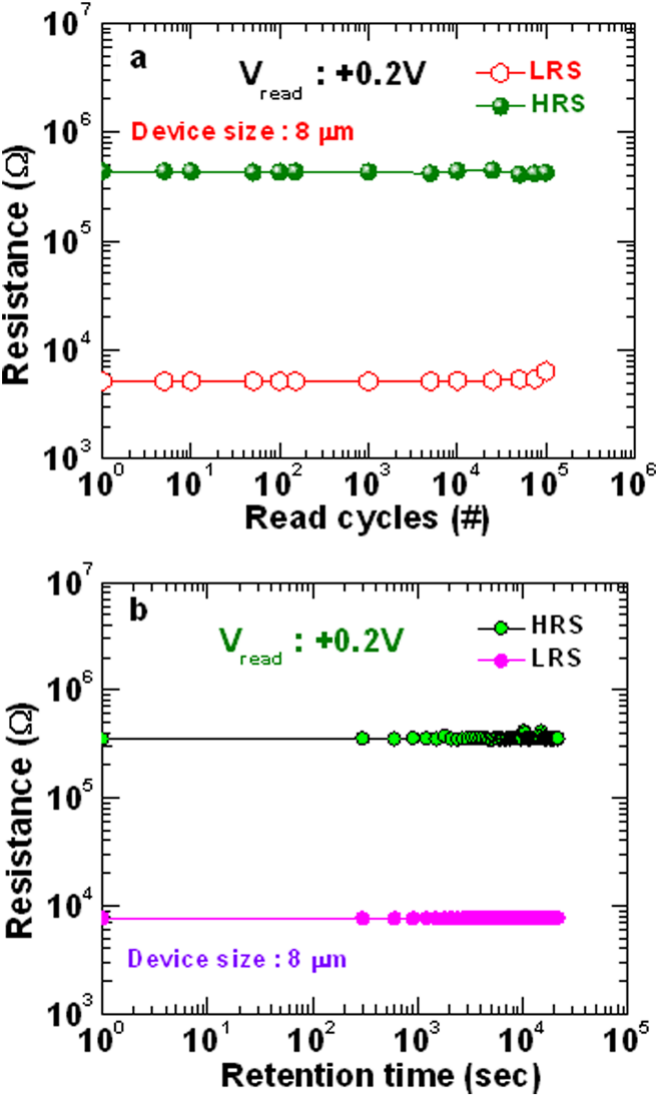
**Read endurance and data retention characteristics. (a)** Long read pulse endurance of >10^5^ cycles and **(b)** long data retention of >3 × 10^4^ s of the 8-μm devices are obtained. The thickness of GdO_x_ film is 17 nm.

Figure 8a represents *I-V* characteristics of successive 90 cycles with good uniformity for the 8-μm devices with a GdO_x_ film of 17 nm. This is also confirmed by current distribution of HRS and LRS at a read voltage (*V*_read_) of +0.2 V, as shown in Figure 8b. The memory devices show an excellent cycle-to-cycle uniformity. The average value (μ) of current in HRS and LRS at a *V*_read_ of +0.2 V are 290 nA and 32.6 μA respectively and standard deviations (σ) are 0.19 and 9.84, respectively. The memory device performs P/E endurance of >100 cycles with a resistance ratio of >5, as shown in Figure 8c. The P/E voltage was +2.5/-2 V and pulse width was 500 μs. The programming and erasing currents were 300 and 500 μA, respectively. Figure 9 shows read endurance and data retention characteristics of the 8-μm devices with a GdO_x_ film of 17 nm. Figure 9a represents long read endurance characteristics of >10^5^ cycles. Stress pulse width was 500 μs. The read pulse width was 10 ms. Both resistance states were read out at +0.2 V. After 10^5^ cycles, good resistance ratio is found to be >100. Figure 9b exhibits good data retention for more than 3 × 10^4^ s with a resistance ratio of >70. Before a data retention test, the device with a size of 8 μm was programmed or erased at a CC of 300 μA. This new RRAM device is very useful for future nanoscale non-volatile memory applications.

## Conclusions

RRAM characteristics by measuring more 50 randomly picked devices in a new Cr/GdO_x_/TiN structure have been investigated. HRTEM images confirm that a GdO_x_ material exists as polycrystalline and thickness of GdO_x_ layer is 17 nm. The GdO_x_ film is also confirmed by EDS and XPS analyses. Large size of the 8-μm devices show better resistive switching characteristics as compared to those small size of the 0.4-μm devices at a CC of 300 μA under low operating voltage of ±4 V, which is due to higher oxygen recombination rate of oxygen with remaining conducting filament in the GdO_x_ film as well as larger TE/GdO_x_ interfacial area. Switching mechanism is based on formation and rupture of the oxygen vacancy conducting filaments through the GdO_x_ grain boundaries. The 8-μm devices show repeatable switching cycles, good device-to-device uniformity, and long read pulse endurance of >10^5^ cycles. Memory device also performs excellent data retention of more than 3 × 10^4^ s with a high resistance ratio of >70. Therefore, the Cr/GdO_x_/TiN RRAM device shows a great potential for future nanoscale non-volatile memory application.
